# Identification of early and intermediate biomarkers for ARDS mortality by multi-omic approaches

**DOI:** 10.1038/s41598-021-98053-1

**Published:** 2021-09-23

**Authors:** S. Y. Liao, N. G. Casanova, C. Bime, S. M. Camp, H. Lynn, Joe G. N. Garcia

**Affiliations:** 1 Department of Medicine, National Jewish Health, Denver, CO USA; 2 The University of Arizona Health Sciences, 1501 N. Campbell Ave, PO Box 245035, Tucson, AZ 85724 USA; 3 Cedars-Sinai Medical Center, Pulmonary Division, Los Angeles, CA USA

**Keywords:** Genetics, Molecular biology, Biomarkers, Diseases, Molecular medicine

## Abstract

The lack of successful clinical trials in acute respiratory distress syndrome (ARDS) has highlighted the unmet need for biomarkers predicting ARDS mortality and for novel therapeutics to reduce ARDS mortality. We utilized a systems biology multi-“omics” approach to identify predictive biomarkers for ARDS mortality. Integrating analyses were designed to differentiate ARDS non-survivors and survivors (568 subjects, 27% overall 28-day mortality) using datasets derived from multiple ‘omics’ studies in a multi-institution ARDS cohort (54% European descent, 40% African descent). ‘Omics’ data was available for each subject and included genome-wide association studies (GWAS, n = 297), RNA sequencing (n = 93), DNA methylation data (n = 61), and selective proteomic network analysis (n = 240). Integration of available “omic” data identified a 9-gene set (*TNPO1, NUP214, HDAC1, HNRNPA1, GATAD2A, FOSB, DDX17, PHF20, CREBBP*) that differentiated ARDS survivors/non-survivors, results that were validated utilizing a longitudinal transcription dataset. Pathway analysis identified TP53-, HDAC1-, TGF-β-, and IL-6-signaling pathways to be associated with ARDS mortality. Predictive biomarker discovery identified transcription levels of the 9-gene set (AUC-0.83) and Day 7 angiopoietin 2 protein levels as potential candidate predictors of ARDS mortality (AUC-0.70). These results underscore the value of utilizing integrated “multi-omics” approaches in underpowered datasets from racially diverse ARDS subjects.

## Introduction

Acute respiratory distress syndrome (ARDS) is a systemic disease which despite increasing health care improvements continues to exhibit an excessive mortality rate of 30–40%^1^. The pathogenesis of ARDS is extremely heterogeneous, involves multiple inciting stimuli, and is influenced by multiple comorbidities and genetic factors. The clinical and biological ARDS heterogeneity contributes to the inability of current clinical severity scoring systems such as the APACHE II score^2^ or the lung injury severity score^3^ to accurately predict ARDS mortality^4^. As clinical factors alone are recognized to poorly predict ARDS outcomes, omics-based biomarkers^5^ offer a potentially unbiased tool for ARDS mortality prediction and may potentially identify specific endotypes and potential therapeutic targets worthy of further investigation. Previous genome-wide association studies (GWAS) identified single nucleotide polymorphisms (SNPs) associated with ARDS susceptibility and mortality which have been summarized in several reviews^5,6^. Multiple transcriptomic, methylation or proteomic studies related to ARDS have been recently summarized^7,8^, however, these studies have largely focused on ARDS susceptibility and a single ‘omics’ approach. In contrast, the potential exists for a multi-omics approach to elucidate pathogenic pathways that enhance understanding of biological pathways and therapeutic target discovery^9^. Systems biology approaches provide a global map of the functional relationships between cellular entities such as genetics, genomics, methylation, and proteomics, and, in the specific case of ARDS, a framework to develop a comprehensive algorithm to identify the molecular patterns associated with ARDS mortality. These findings may guide the utilization of molecular signatures to inform clinical decision-making in ARDS and ultimately identify novel therapeutic targets.

To address the unmet need for novel ARDS biomarkers, an integrated “multi-omics” approach was utilized in a racially-diverse ARDS cohort encompassing individually- underpowered ARDS GWAS, RNA transcription, and DNA methylation datasets with a goal to identify early and intermediate predictive biomarkers of ARDS mortality. These analyses yielded a 9-gene set that differentiated ARDS survivors from non-survivors and prioritized a biologic pathway list which included the p53-, HDAC-, TGF-β-, and IL-6-signaling pathways, results validated utilizing a longitudinal transcription dataset. Predictive biomarker discovery revealed that both transcription levels of the 9-gene set (*TNPO1, NUP214, HDAC1, HNRNPA1, GATAD2A, FOSB, DDX17, PHF20, CREBBP*) and Day 7 angiopoietin-2 protein levels significantly predict ARDS mortality. The application of a multi-omics integrated prediction model for ARDS mortality may be of substantial utility in the design of future ARDS clinical trials.

## Results

### Study population characteristics

**Figure 1:**
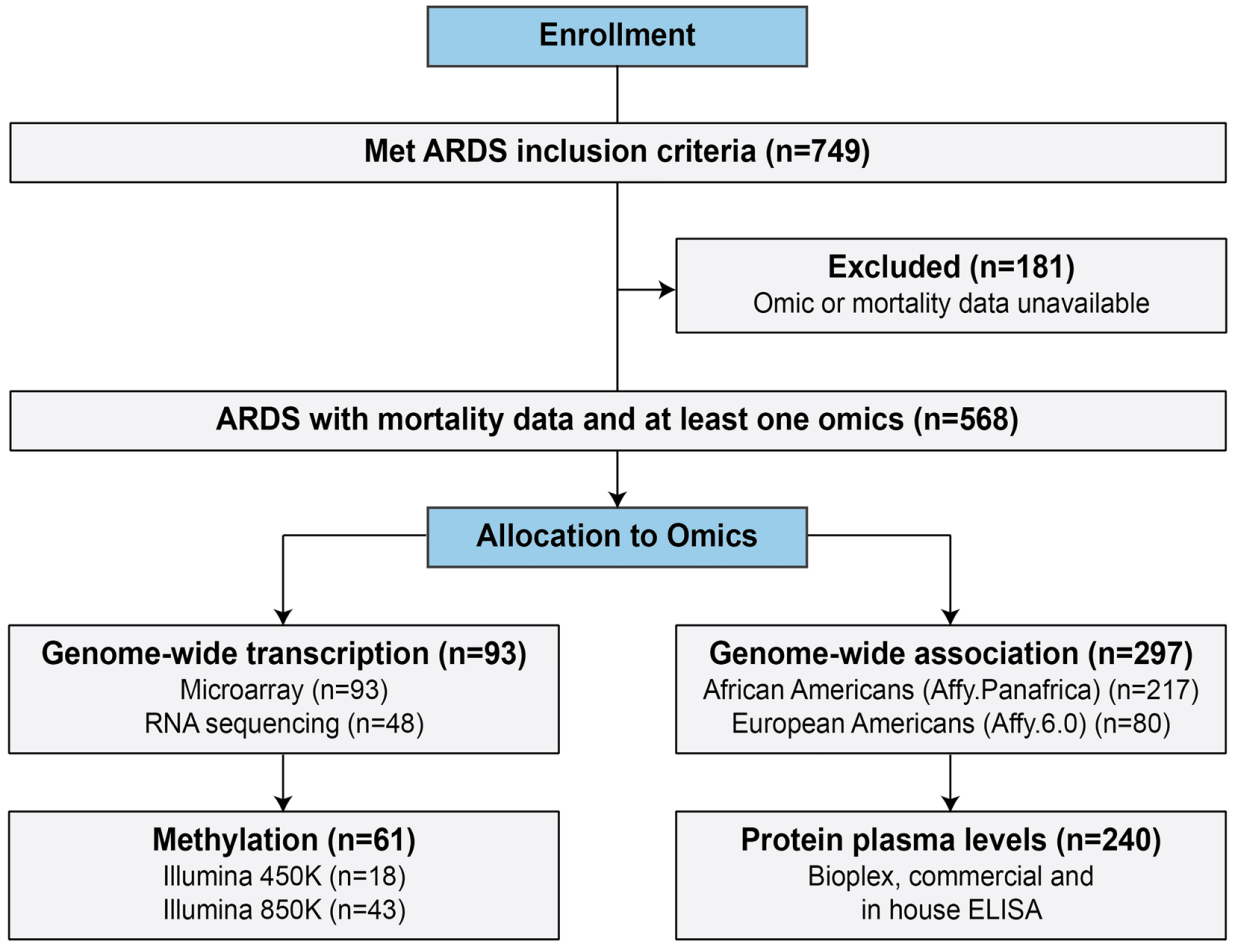
Flowchart of total study enrollment and subjects included in each of the “omics” platforms.

A diverse cohort of 568 subjects (mean age of 53 ± 15 years, male 55%, 40% self-reported Blacks) with an overall day 28 mortality rate of 27% was included in the analysis (Fig. 1). Table 1 contains the basic cohort characteristics stratified by each ‘omic’ approach utilized (with overlap). Supplemental Figure S1 depicts the Venn diagram of overlapping ‘omics’ data from the study population. The majority of cohort subjects had genotyping and protein data available.

**Table 1:** Basic characteristics for the study population stratified by each ‘omics’ type.

Basic characteristics	Overall N = 568	Genetics N = 297	Transcriptions^a^ N = 93	Methylations N = 61	Protein N = 240
Age: years, mean ± SD	52.5 ± 15.6	54.3 ± 16.3	50.1 ± 14.5	53.2 ± 16.1	50.4 ± 14.3
Gender: male, n (%)	312 (54.9)	181 (60.9	46 (49.5)	35 (57.4)	124 (51.7)
Race: White	305 (53.7)	77 (25.9)	64 (68.8)	31 (50.8)	175 (72.9)
Race: Black	227 (40.0)	217 (73.1)	6 (6.5)	22 (36.1)	40 (16.7)
Race: Other	36 (6.3)	3 (1.0)	23 (24.7)	8 (13.1)	25 (10.4)
Pneumonia, n (%)	150 (26.4)	24 (8.1)	45 (48.4)	9 (14.8)	126 (52.5)
Death, n (%)	152 (26.8)	86 (29.0)	35 (37.6)	26 (42.6)	53 (22.1)
ICU stay length, days, mean ± SD	29.0 ± 21.7	30.6 ± 21.5	31.0 ± 19.5	25.2 ± 21.0	26.9 ± 21.8

*SD* standard deviation, *ICU* intensive care unit.

^a^Transcriptions including RNA microarray and RNA-sequencing data.

### Genome-wide SNP analysis and identification of genes associated with ARDS mortality

GWAS association analysis of SNPs failed to identify a SNP that passed genome wide multiple testing correction threshold (p < 5 × 10^–8^) associated with ARDS mortality in either study population (African or European descent). In addition, gene-based analysis (VEGAS2, total of 23,678 genes), also failed to genes significantly associated with ARDS mortality i.e. p < 2.11 × 10^–6^. The top genes (*ASB3*, *GPR75*, *MIR3682*, *ERLEC1)* were each located on chromosome 2 with p < 5 × 10^–4^, 28 genes exhibited a p < 5 × 10^–3^ and 601 genes a p < 5 × 10^–2^ (Supplemental Table S1).

### Genome-wide RNA transcriptome analysis of genes associated with ARDS mortality

RNA sequencing data from ARDS survivors and non-survivors (n = 48) failed to identify a significant DEG (Supplemental Table S2) with the top 3 genes in non-survivors *(*upregulated *PLK5*, *ARHGEF33*, downregulated *ACO092329.3)* exhibiting p < 10^–4^.

### Genome-wide gene methylation analysis associated with ARDS mortality

We next analyzed genome-wide DNA methylation from ARDS survivors and non-survivors (n = 60) utilizing the combined 450 k and 850 k CpG EPIC array datasets. With an FDR of 0.05, no significant CpG site was identified through the probe-wised differential analysis. The top two probes were cg19741456 in *FBXO6* and cg25191743 in *MAP3K14* (raw p < 5 × 10^–5^). The top results are shown in Supplemental Table S3.

### Analysis of potential ARDS plasma biomarkers

We interrogated 11 plasma proteins previously implicated as potential ARDS biomarkers^6^ with levels measured on entry to ICU (baseline at Day 0) and at Day 7 for survivors. No baseline protein level was associated with ARDS mortality whereas Day 7 plasma measurements of Ang2 protein were significantly associated with ARDS mortality. Each 1 ng/mL increase in plasma Ang2 protein at Day 7, produced an 8% increase in odds of mortality (p = 4.7 × 10^–6^). We included analysis for protein measured on baseline and Day 7 individually to avoid the correlation between measurements on the same subject. A full result of the logistic regression is shown in Supplemental Table S4.

### Candidate gene analyses

While no candidate gene reached the statistical threshold defined by FDR adjusted p-value < 0.05 in any ‘omics’ analysis, several candidate genes exhibited raw p-values < 0.05. This included differential methylation of cg18537894 in *ADIPOQ* (raw p-value = 0.02), upregulated *NAMPT* gene expression in non-survivors compared to the survivors (log fold change = 1.41, raw p = 0.01) and upregulated *EGLN1* gene expression in non-survivors compared to survivors (log fold change = 0.65, raw p = 0.03) with significant differential *EGLN1* methylations (cg20682143; cg21875980; cg18979762, raw p = 0.02). No significant associations of *FER, IL1B, or TNF* were found in any ‘omics’ types. The results were summarized in Table 2.

**Table 2:** Summary of the results from the candidate gene analysis.

Data	GWAS data	RNA sequencing		Methylation data from 450 and 850 k EPIC platforms			Protein level on baseline and Day 7	
Analysis	Gene-based analysis	Differential expression analysis		Differential methylation analysis			Logistic regression	
Gene name	p-value	LogFC	p-value	Probe	Log FC	p-value*	Estimate (p-value) Day 0	Estimate (p-value) Day 7
ANGPT2	0.98	−0.04	0.78				−0.01 (0.32)	**0.08 (4.7e−06)**
IL1R2	0.91	1.50	0.11				−9.33e−07 (0.84)	1.62e−05 (0.16)
ADIPOQ	0.75			cg18537894	0.17	0.02		
NAMPT	0.66	1.41	0.01				4.09e−04 (0.79)	−3.1e−04 (0.81)
FER	0.44	−0.03	0.88					
TNF	0.46	0.16	0.67					
IL1B	0.92	0.59	0.30				2.21e−03 (0.32)	−2.5e−03 (0.48)
EGLN1	0.34	0.65	0.03	cg20682143 cg21875980 cg18979762	0.25 −0.65 0.11	0.02 0.02 0.05		
IL-6	0.92	−0.60	0.07				−4.51e−05 (0.65)	0.001 (0.35)
IL-8	0.73	0.06	0.93				−0.00021 (0.35)	0.0008 (0.06)
VEGF	0.44	−0.01	0.96	cg08826863	−0.21	0.04	−0.00015 (0.93)	0.00017 (0.53)
MIF	0.04	−0.26	0.06				−6.12e−05 (0.32)	0.0023 (0.17)
S1PR3	< 0.01	−0.24	0.35				0.00017 (0.18)	4.7e−05 (0.20)
HMGB1	0.73	−0.32	0.01				−0.00071 (0.23)	−5.11e−05 (0.80)

*GWAS* genome-wide association study, *Chr* chromosome, *LogFC* log of the fold change.

Bold: significant after corrected for multiple comparisons.

*Only reported the methylation results with raw p-values < 0.05.

### Multi-omic integration and pathway analysis

**Figure 2:**
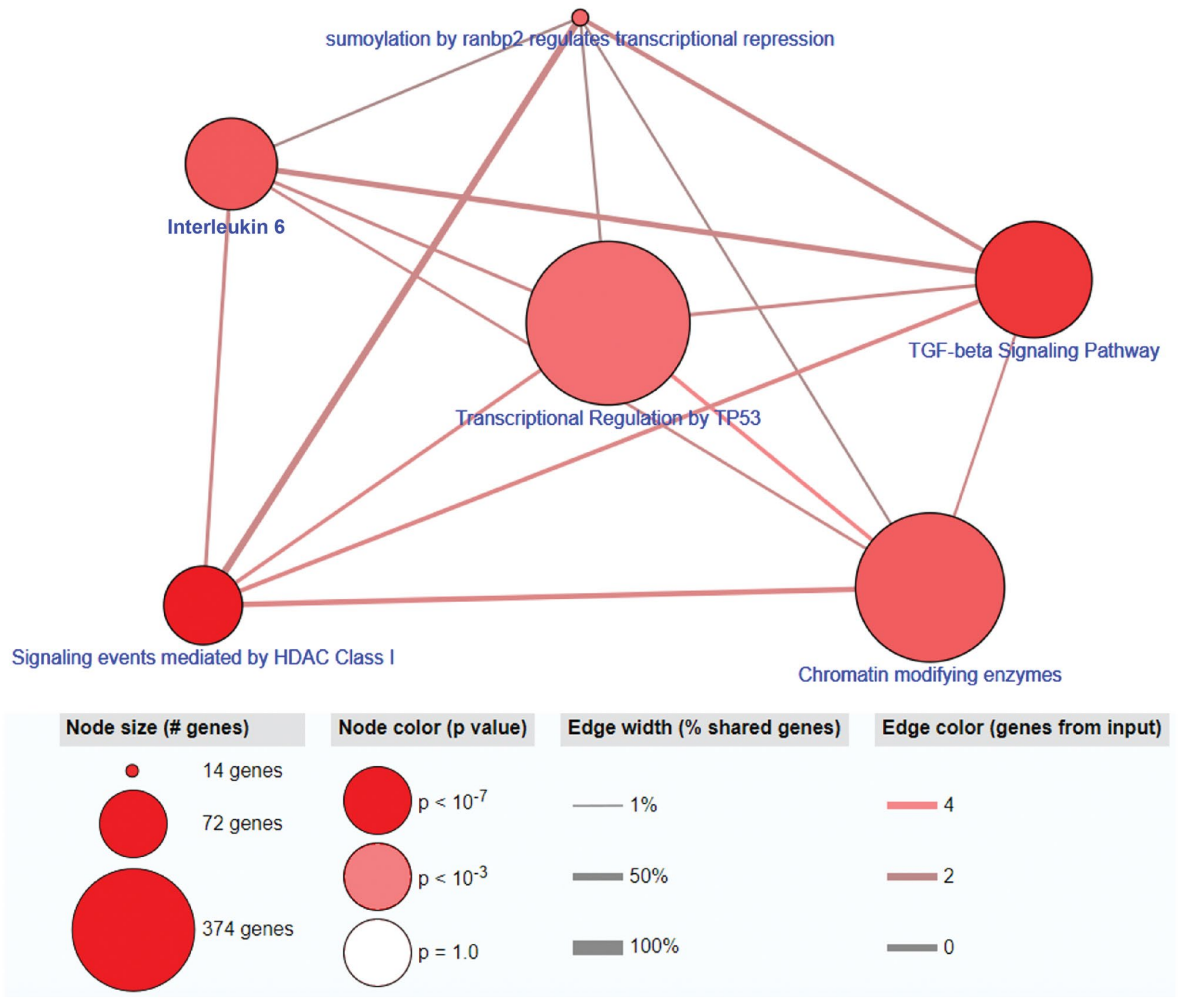
Relationship between top identified pathways associated with ARDS mortality using the *Network approach* and ConsensusPathDB^26^.

Using the *Network approach* and the dense module search method^10^, 9082 nodes (genes) and 51,871 edges (protein–protein interactions or PPIs) were included in the final network resulting in a total of 5854 modules identified. The top 0.1% of modules included six modules and 25 unique genes with 9 of 25 unique genes also differentially methylated (raw p-value < 0.05): *TNPO1, NUP214, HDAC1, HNRNPA1, GATAD2A, FOSB, DDX17, PHF20,* and *CREBBP*. The results of these 9 genes in each omic type are shown in Supplemental Table S5. Further evaluation of this 9 differentiating gene-set, revealed 87 pathways with a q-value < 0.05 with the top unique pathways consisting of ‘HDAC class I signaling’, ‘TGF-β signaling pathway’, ‘IL-6 signaling pathway’, ‘Chromatin-modifying enzymes’, ‘Sumoylation by ranbp2 regulates transcriptional repression’ and ‘Transcriptional regulation by TP53’. These pathways are listed in Table 3 and Fig. 2 and the full list is provided in Supplemental Table S6.

**Table 3:** Significant pathways associated with ARDS mortality using the *Network approach*.

Pathway name	Set size	Candidate^a^	q-value	Source
Signaling events mediated by HDAC class I	56	4 (7.1%)	3.45E−06	PID
TGF-beta signaling pathway	132	4 (3.0%)	5.55E−05	Wikipathways
IL-6 pathway	74	3 (4.1%)	0.000327	NetPath
Chromatin modifying enzymes	272	4 (1.5%)	0.000327	Reactome
Sumoylation by ranbp2 regulates transcriptional repression	14	2 (14.3%)	0.000497	BioCarta
Transcriptional regulation by TP53	374	4 (1.1%)	0.000602	Reactome

^a^Numbers of genes in the pathway are from the 9 genes identified through the Network approach.

Using the *Overlap gene approach*, we identified an overlap gene list consisting of ten genes (*OCIAD2, MLST8, TOP3A, ZC3H8, C4orf3, PIP5K1A, PLD5, TNFRSF19, C14orf93, RUNX3)* with the seven most significant pathways (q-value < 0.05) being ‘RNA polymerase II transcription’, ‘Regulation of TP53 activity’, ‘PIP3 activates AKT signaling’, ‘Intracellular signaling by second messengers’, ‘Generic transcription pathway’, and ‘Transcriptional regulation by TP53’ (Table 4). Thus, there was a significant overlap between pathways (but not genes) identified utilizing both the *Overlap* and *Network* pathway approaches.

**Table 4:** Significant pathways associated with ARDS mortality using the *Overlap gene approach*.

Pathway name	set size	# Candidates^a^	q-value	Source
RNA polymerase II transcription	1236	4 (0.3%)	0.00598	Reactome
Gene expression (transcription)	1373	4 (0.3%)	0.00598	Reactome
Regulation of TP53 activity	162	2 (1.2%)	0.00598	Reactome
PIP3 activates AKT signaling	214	2 (0.9%)	0.00775	Reactome
Intracellular signaling by second messengers	245	2 (0.8%)	0.00808	Reactome
Generic transcription pathway	1107	3 (0.3%)	0.0129	Reactome
Transcriptional regulation by TP53	374	2 (0.5%)	0.0129	Reactome

^a^Numbers of genes in the pathway are from the 10 genes identified through the Overlap gene approach.

### Validation using longitudinal transcription analysis

**Figure 3:**
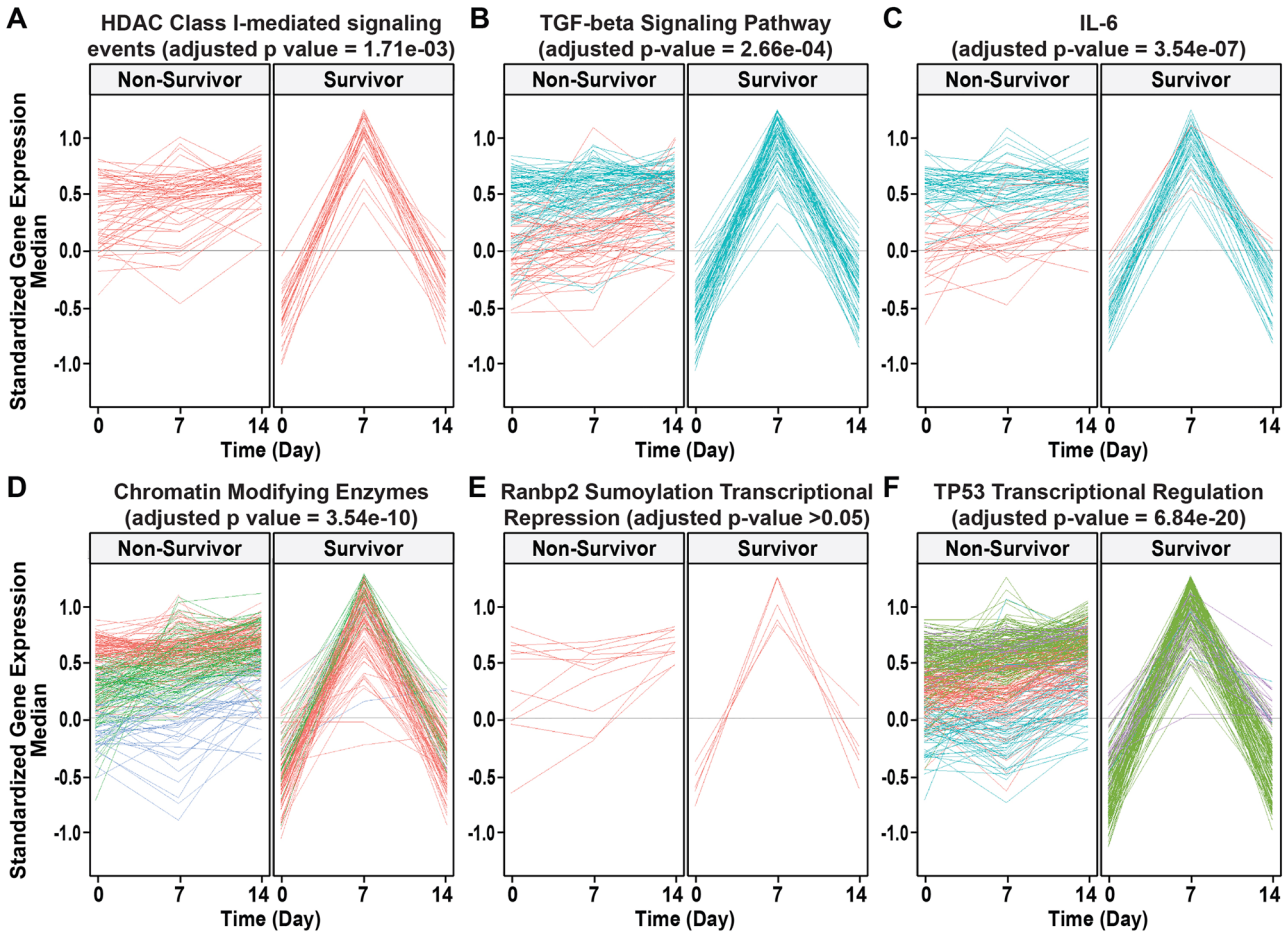
Shown are the changes in median pathway expression (gene set) between ARDS survivors and non-survivors for specifically identified pathways including HDAC (**A**), TGFβ (**B**), IL-6 **(C**), Chromatin (**D**), sumoylation (**E**), and TP53 (**F**). Each line represents the gene expression of a pathway gene. The color of the line represented the cluster of the pattern, a line within the same group with the same color means the patterns of change for those genes are similar. For example, in the TGF-β signaling pathway, the gene expression change was homogeneous in the survivor group (all blue) while two clusters of patterns existed in the non-survivor group (blue and red). The adjusted p-value represented the significance of those patterns changes between groups. The color reflects the pattern of the gene expression change within that specific pathway and group (not use to compare groups or pathways). The red only lines in the HDAC pathway indicate non-survivor and survivor gene pattern change have only one pattern whereas in the TGFβ pathway non-survivors have two patterns (red and blue) but survivors only one pattern.

Temporal gene/pathway expression was significantly different between ARDS survivors and non-survivors in five out of the six tested pathways (“sumoylation by ranbp2 regulates transcriptional repression” the exception). The Transcriptional Regulation by TP53 pathway was the most significant pathway (adjusted p-value = 6.84e−20) with median gene set expression (median of standardized gene expression) elevated at baseline, remaining elevated at Day 14 in non-survivors. In ARDS survivors, the median gene set expression was lower than non-survivors at baseline, elevated at Day 7 but returned to baseline values by Day 14. In addition, the patterns of pathway expression were reproducibly homogeneous in ARDS survivors when compared to non-survivors. Statistical differences in survivor and non-survivor patterns and in median gene expression changes over time are shown in Fig. 3.

### Mortality prediction model development

**Figure 4:**
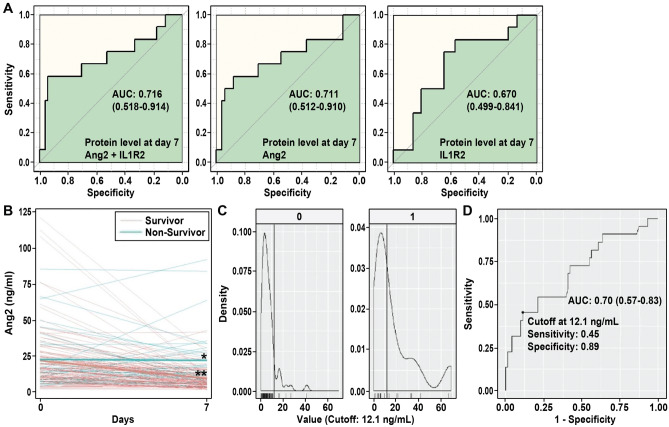
**(A)** AUC plots of the prediction model using Day 7 Ang2 and IL1R2 (alone and combined). Day 7 Ang2 alone has significant discrimination between ARDS survivor/non-survivor groups. (**B)** Mean concentration of *Ang2* in Day 0 (baseline) and Day 7 between ARDS survivor/non-survivor groups showed the mean concentration of Ang2 decrease with time in the survivor group. * Mean concentration for death; ** Mean concentration for survivors. (**C)** Independent variable optimized cutoff and distribution by class (0 = alive, 1 = death); cutoff concentration of 12.1 ng/mL. (**D)** ROC curve, sensitivity and specificity using Day 7 Ang2 protein level to predict ARDS mortality which showed with the cutoff concentration of 12.1 ng/mL, the specificity for ARDS mortality is high at 0.89.

Supplemental Table S5 and Table 2 summarize the overall results (detail result in each molecular level) for the multi-omics analysis with p-values for novel genes identified through the multi-omics approach and for candidate genes selected from the publicly available literature. The transcription prediction model included the nine genes in the logistic regression model with the derived AUC from the testing set (baseline RNA microarray) at 0.83 (90% C.I. 0.62–1.00). For the protein prediction model, the AUC of the prediction model, using baseline protein levels as predictors, was poor (< 0.5). However, when Day 7 proteins were used as candidate predictors, Ang-2 and IL-1R2 were predictors for ARDS mortality through the stepwise method with derived AUC of 0.72 (90% C.I. 0.52–0.91). We next focused on 172 subjects with both Day 7 Ang2 and IL-1R2 protein measurements and assessed AUCs of Ang2 and IL-1R2 which demonstrated that Ang2 alone (AUC 0.71, 90% C.I. 0.51–0.91) exhibited a higher AUC compared to IL-1R2 alone and was comparable to both Ang2 and IL-1R2 as predictors (Fig. 4A). Figure 4B shows the trend of Ang2 protein level changes in survivor and non-survivor groups with median Ang2 concentrations similar at baseline between the two groups. In contrast, Day 7 Ang2 levels decreased in survivors but remained elevated in non-survivors with the optimized cutoff point for ARDS mortality prediction shown in Fig. 4C. Using a cutoff point of 12.1 ng/ml, the sensitivity and specificity of Ang2 as a test for ARDS mortality were 0.45 and 0.89, respectively. The AUC of the validation cohort (Fig. 4D) consisting of 93 subjects with Ang2 measurement was 0.70 (90% C.I. 0.57–0.83).

## Discussion

The present study utilized multi-omic approaches to identify potential early and intermediate predictive indicators of and contributors to ARDS mortality which include: (i) a 9-gene expression set, (ii) plasma Ang2 protein levels, and iii) the p53-, TGFβ-, and IL-6 signaling pathways. Through the integration of genetics, transcriptomics, methylation, and protein–protein interaction, we identified novel genetic networks that potentially contribute to the pathogenesis of ARDS mortality. These genes and pathways were validated using longitudinal transcriptional data further strengthening the prospects of their involvement in ARDS pathobiology and mortality. A strength of our analyses was the utilization of a racially- and gender-diverse ARDS cohort exhibiting multiple ARDS-inciting causes. Whereas traditional approaches have individually evaluated each ‘omic” dataset (genotype, transcription, methylation, protein), we chose to integrate underpowered genotyping, microarray, and sequencing datasets to embrace the relationship between different omics largely lost by traditional approaches. Genotypes and methylation data may alter gene transcription and result in altered protein expression and, therefore, may miss important biological information. Our systems biology studies indicate that combining several small, albeit woefully underpowered ‘omic’ studies, may increase the power of identifying significant and important biological pathways and biomarkers that can predict/contribute to ARDS mortality.

At the single gene level, nine genes were associated with ARDS mortality through a systems biology approach including *HDAC1*, encoding a histone deacetylase (HDAC) previously linked to acute inflammatory disorders^11–13^ including acute lung injury with HDAC inhibitors shown to prevent endothelial hyperpermeability^14^, the major pathophysiological defect in acute lung injury^15–17^. HDAC inhibitors are often included in lists of potential therapeutic ARDS approaches^18^. Another 9-gene set member is *HNRNPA1* which encodes heterogeneous nuclear ribonucleoprotein A, a recognized regulator of alternative splicing processes including alternate splicing of *MYLK*^19^, a well-described ARDS candidate gene^20^ and via its encoded protein, non-muscle myosin light chain kinase isoform (nmMLCK), a key regulator of lung vascular integrity^21^. *FOSB* is involved in ventilator-induced lung injury (VILI) via evoked *AP-1* binding and gene expression^22^, increased ROS burden, and VILI outcomes^23^. Transportin 1 (*TNPO1*) is a protein involved in importing proteins into the nucleus with prominent targets being RNA-binding proteins including HNRNPA1, the same gene identified by our ‘omics’ approach^24^. The PHF20 *(PHF20)* protein regulates p53 signaling and epigenetic regulation by chromatin regulation/histone deacetylases^25^, as does CREBBP protein a histone acetyltransferase and transcriptional coactivator^26^, findings in sync with the 9-gene set, DNA methylation, and protein datasets we analyzed. Less information is available for proteins encoded by *GATAD2A*, *DDX17*, *NUP214* although they appear to share estrogen and androgen receptor regulatory properties^27–30^.

*Ang2 (angiopoi*etin 2) is a ligand of endothelial receptor Tie2 and a key mediator of pulmonary vascular permeability and Day *7* Ang2 protein levels were an intermediate biomarker for ARDS mortality, a finding consistent with a recent meta-analysis that higher plasma Ang2 levels are predictive of mortality in both ARDS and at-risk patients^31–34^. Our studies showed that Day 7 Ang2 has a better predictive value compared to baseline Ang2 which implies that Ang2 may respond to endothelial damages from ARDS in a later phase. In addition to outlining the relationship between mortality and the level of Ang2 at different stages, our study further provides a cutoff point of the Ang2 concentration for mortality prediction. We identified that Day 7 Ang2 level of 12.1 ng/mL can be used as a highly specific tool for the ARDS mortality and may offer potentially clinically useful information and promote therapies that target Ang2. IL-1R2 (IL-1 Receptor Type 2), which regulates the proinflammatory activities of IL-1β, is an ARDS biomarker^35^ with *IL1R2* gene expression upregulated in ARDS patients compared to controls. We found plasma IL1R2 protein levels between Days 7 and 14 higher in ARDS non-survivors compared to survivors, a trend not seen at days 0 to 3. The results support our findings that in addition to Ang2, Day 7 IL-1R2 levels, but not baseline IL1R2 levels, is a good biomarker for ARDS mortality prediction.

At the pathway level, five of six pathways identified by our multi-omic approaches exhibited similar expression patterns over time and significantly distinguished ARDS survivors from non-survivors. The pathway expression patterns in survivors were low on Day 0 (baseline), while elevated at Day 7 and returned to baseline values on Day 14. In contrast, pathway expression in non-survivors was elevated at Day 0 compared to survivors, continued to rise at Day 7, and remained elevated at Day 14, results that suggest that activation these of pathways contributes to ARDS mortality. The TP53 pathway was the most significant pathway identified (via both *Network* and *Overlap* approaches) and centers on the functional strong anti-inflammatory role of p53 as a tumor suppressor protein. Inflammation involving NFkB activation reduces p53 function and p53 activation is an inflammation-suppressing response to reduce unchecked vascular leakage^36^. In contrast, HDAC pathway^18^ and TGF-β activation may increase endothelial permeability and play important role in lung fluid balance in ARDS^37,38^. We also found genes in the well-known pro-inflammatory IL-6 pathway^39^, were highly expressed in ARDS non-survivors throughout the whole 14 days, a finding in sync with studies demonstrating persistently elevated plasma or BAL IL-6 levels in ARDS non-survivors^40^ predicting poor ARDS outcomes (e.g., prolonged mechanical ventilation, organ dysfunction, and mortality)^41–45^. Taken together, except for *ANGPT2* (the protein name is Ang2), none of the candidate genes (*IL1R2, ADIPOQ, NAMPT, EGLN1, FER, IL1B*, *TNF*, *IL-6*, *IL-8 (CXCL8), VEGF (VEGFA), MIF, S1PR3,* or *HMGB1)* were significant in our multi-omic analysis of ARDS mortality. The findings are not surprising since mortality is a complicated biologic process and is not highly unlikely to be driven by a single gene. Nevertheless, our pathway analysis implicated several candidate genes in pathways mortality affecting ARDS such as HDAC- and IL-6-signal pathways.

Clearly, our study has several limitations, the foremost limitation being that despite an excellent number of ARDS subjects, few subjects had available data from more than three omic types and limited time points availability, thereby straining the ability to expand the longitudinal analysis. As few subjects had multiple omics measured at the same time, this limited the robustness of our analytical methodologies. For example, a more complete and integrative predictive model using all omics data simultaneously cannot be applied in our dataset. In addition, our mortality outcome is a categorical outcome because we did not collect the day of the death which may reduce our power and the information derived from the analysis. An inherent limitation is that each “omic” data approach has advantages and disadvantages depending on study aims. For example, GWAS genotypes are easily measured and offer disease susceptibility information but cannot reflect the influence of environmental factors and therefore are limited in ARDS mortality associations. Gene expression and DNA methylation together reflect both genetic and environmental factors and are good ‘omic’ types for mechanism investigation, however, these require more sophisticated sample processing to obtain the data and due to high cost, have much smaller datasets. Protein biomarkers analytics is a good omic platform, however, as protein levels are affected by genetics, transcriptomics, methylation, and post-translational modification, it is difficult to explore the underlying biological mechanism for ARDS mortality with this platform alone. Additional limitations of our work were the diversity in platforms utilized for our genetic, genomic, and methylation studies including the varying time horizons with limited overlapping specimens, decreasing our statistical power to identify predictive biomarkers. Clearly, future well-designed prospective validation cohorts and timely sampling and analysis will obviate a number of these limitations.

In summary, a well-characterized, diverse ARDS cohort was utilized to assess the potential for a unique multi-omics approach to address the unmet need for tools that allow the stratification of ARDS subjects for clinical trials. This “multi-omics” approach, integrating several ‘omics’ data types from individually underpowered ARDS GWAS, RNA transcription, and DNA methylation datasets, identified a 9-gene set and specific signaling pathways that differentiated ARDS survivors from non-survivors, results validated utilizing a longitudinal transcription dataset. The prioritized list of genes and p53-, HDAC-, TGF-β-, and IL-6-signaling pathways exhibit high biological plausibility to serve as early and intermediate predictive biomarkers and pathways for ARDS mortality. Although further studies are needed, these results suggest that the additional application of a multi-omics integrated prediction model for ARDS mortality may be of utility in future ARDS clinical trial design.

## Methods

### Study population

A total of 749 subjects, 18 years and older, have a diagnosis of ARDS established according to the diagnostic criteria per the American-European Consensus Conference (AECC)^46^ or the Berlin definition^47^. All the experimental protocols were performed in accordance with guidelines and regulations and approved by institutional review boards (IRB) at the following institutions: the University of Arizona Health Sciences (IRB#1312168664R001), the University of Illinois at Chicago (IRB# 20120192), and the University of Chicago (IRB#15194A), patient enrollment occurred from 2007–2018. Specimens from patients enrolled in the NIH-funded Fluid and Catheter Treatment Trial (FACTT) study were also included^48^. Enrollment included subjects admitted to the intensive care units with confirmed ARDS. Written consent was obtained for all the participants. Among the 749 subjects, a total of 568 ARDS subjects with mortality information (mortality at day 28) and with at least one type of ‘omics’ data available were included in the final analysis (Fig. 1).

### Genome-wide genotyping

Genome-wide genotyping was performed using two platforms for two distinct populations based on race: Affymetrix SNP 6.0 (Thermo Fisher Scientific, Santa Clara, CA) for the European-origin populations and Affymetrix Pan-African array (Thermo Fisher Scientific, Santa Clara, CA) for the African descent individuals. Subjects with a call rate of less than 98% were removed from the analysis. SNPs were removed if failing the Hardy–Weinberg equilibrium test (p-value < 10^–6^) or a call rate of less than 98%. After the quality control steps, a total of 297 ARDS subjects: 80 European-origin subjects with 905,043 SNPs, and 217 African Americans with 146,595 SNPs, were included in the analysis.

### Genome-wide transcription profiling

Genome-wide transcription data were obtained using two gene expression platforms, RNA microarrays, and sequencing. RNA was available for a total of 93 participants with a total of 136 samples at different time points. Among the 93 participants, 7 participants had two baseline RNA samples (sent for both RNA-sequencing and RNA-microarray), 8 participants had RNA samples on Day 7 after enrollment and 28 participants had RNA samples on Day 14 after enrollment. Peripheral blood mononuclear cell (PBMC) isolation was performed using the Ficoll-Paque™ (Sigma-Aldrich) method^49^. Total RNA was isolated from PBMCs using RNAeasy MiniKit Qiagen™ following the manufacturer’s protocol. RNA concentration and quality (RIN > 7) were assayed by Nanodrop™ (Thermo Fisher) and 2100 Bioanalyzer RNA™ (Agilent). A total of 52 baseline samples, 8 Day 7 samples, and 28 Day 14 samples were sent for transcription profiling using RNA microarray Affymetrix GeneChip Human Gene 2.0 ST Array (Thermo Fisher). A detailed protocol and quality control procedure for RNA microarray profiling has been previously reported^50^. The gene expression levels were normalized with a log transformation. After quality control, a total of 17,641 probes were included in the downstream analysis.

Samples of 1–2 μg total RNA from a cohort of 48 ARDS patients were sent for transcription profiling via RNA sequencing with the RNA concentration and quality (RIN > 7) assessed by Nanodrop™ (Thermo Fisher) and corroborated by Bioanalyzer™ (Agilent). To prepare the sequencing library, total RNA was enriched by oligo (dT) magnetic beads (rRNA removed). The RNA-seq library preparation was performed using KAPA Stranded RNA-Seq Library Prep Kit (Illumina). The completed libraries were qualified with Agilent 2100 Bioanalyzer and quantified by the absolute quantification qPCR method. To sequence the libraries on the Illumina HiSeq 4000 instrument, the barcoded libraries were captured on the Illumina flow cell, amplified in situ, and subsequently sequenced for 150 cycles for both ends on the Illumina HiSeq instrument. R package *Tximport* was used to transform the raw counts from the transcripts into gene counts. Genes with low expression defined by low count (< 6) were removed, a total of 18,652 genes were included in the final analysis.

### Genome-wide DNA methylation profiling

High-quality DNA from PBMCs was available from 61 ARDS subjects for DNA methylation analysis as previously described^51,52^. Additional protocol details are available on the manufacture’s website (Bisulfite conversion Qiagen, hybridization, and Infinium Methylation Assay Illumina). CpG sites were quantitatively assessed utilizing two Methylation array platforms. The initial 18 subjects were assessed by HumanMethylation 450 K BeadChip (Illumina). Subsequently, DNA from a cohort of 43 ARDS subjects was assessed utilizing the MethylationEPIC 850 K BeadChip array (Illumina). Poor quality samples were excluded using a detection p-value cutoff greater than 0.05 using the R package *minifi*. After quality controls, data from the two assays involving 61 subjects were pooled for analysis. Methylation levels were represented as M-values which were calculated as log 2 (methylated/unmethylated) and were used in downstream analyses.

### Plasma protein levels

Plasma biomarker studies were focused on 11 selected proteins: eNAMPT, IL-1β, IL-1R2, IL-6, IL-8, VEGF, Ang2, MIF, S1PR3, RAGE, and HMGB1 with plasma levels measured using Bio-Rad (Hercules, CA), detailed in prior reports^53,54^. In brief, whole blood was collected in EDTA-treated tubes, centrifuged within 1 h from sample collection (2000 × *g* for 20 min, RCF), and stored at − 80 °C. Plasma concentrations of three biomarkers (IL-6, IL-8, IL-1β) were measured in duplicate using a custom Bio-Plex Pro Human Cytokine 5-plex immunoassay (Bio-Rad, Hercules, CA) and Bio-Plex MAGPIX instrument following the manufacturer’s guidelines. Enzyme-linked immunosorbent assay (ELISA) techniques were utilized to quantify plasma levels of eNAMPT (an internally developed ELISA)^54^, MIF (R&D System®, Minneapolis, MN), and Ang-2 (R&D System^®^, Minneapolis, MN) using commercially-available ELISAs according to the manufacturer’s instructions as previously described^53^. Undetectable protein levels were assigned as zero. A total of 240 subjects with at least one protein level measured were included in our analysis.

### Analytical approach and strategies

We first performed the analysis to identify the biomarkers that can differentiate ARDS non-survivor vs. survivor in each single omics platform independently. Second, we integrated multiple omics types to identify important candidate biomarkers and performed a pathway analysis. Finally, we developed a prediction model of ARDS mortality. An overview of the analysis flow is shown in Supplemental Figure S2.

### Genome-wide association analysis

SNP level analysis was performed using PLINK^55^ with a separate analysis of the African-Descent and European-Descent cohorts. First, the principal components (PCs) for population stratification were calculated and included in the first two PCs in the additive regression model. After obtaining individual p-values for each SNP, a gene-based analysis was performed using the method of versatile gene-based association study-2 (VEGAS2) to incorporate genes and accounts for linkage disequilibrium between markers by using simulations from the multivariate normal distribution^56^. To map identified SNPs to their related gene, the 1000 K Genome with African and European datasets were used as reference panels and the gene region was defined as + /− 50 k base pairs. Due to the distinct ethnicity and different platforms, gene-based analyses were conducted in each ethnicity independently and a meta-analysis was then performed to combine the p-values using Fisher’s method.

### Differential expression gene analysis

Differential expression gene (DEG) analysis was performed using the RNA sequencing data and the R package *limma*^57^. We normalized the raw RNA sequencing count data using variance stabilization transformation (VST)^58^. The significant DEG level was defined as the false-discovery rate (FDR) adjusted p < 0.05.

### Differential methylation analysis

Combining data from the 450 K BeadChip and MethylationEPIC BeadChip (Illumina) was used to stratify quantiles for data normalization. Probes were filtered for removal if failing in one or more samples or on the sex chromosome. The methylation data were normalized using the subset quantile normalization^59^. M values were obtained for the probe-wised differential analysis. The analysis was conducted using the R package *minifi*^60^. The threshold for the significance was the FDR adjusted P < 0.05.

### Plasma protein analysis

Logistic regression analysis was used to assess the association between protein levels and ARDS mortality. Age and gender were included in the model as covariates. Associations were tested using the selected 11 protein levels^6^ measured on baseline and Day 7 respectively instead of including both baseline and Day 7 protein levels to avoid the correlation between these two measures in the same individual.

### Candidate gene analysis

In addition to the genome-wide approach mentioned above, individual candidate genes, selected from previous publications^6^, were tested via a multi-omics approach. The candidate genes included were *ANGPT2* (the protein name is Ang2), *IL1R2, ADIPOQ, NAMPT, EGLN1, FER, IL1B*, *TNF*, *IL-6*, *IL-8 (CXCL8), VEGF (VEGFA), MIF, S1PR3,* and *HMGB1*^54,61,62^.

### Multi-omic integration and pathway analysis

Two approaches were selected to achieve multi-omic integration and subsequent pathway analysis: the *Network approach* and the *Overlap gene approach*. Results from each approach were then compared and overlapping genes/pathways were identified that were associated with the ARDS mortality.

#### Network approach

The method of edge-weighted dense module search was first used to identify potential genes associated with ARDS mortality^10^. The R package, *dmGWAS_3.0,* was used to build the network by combining GWAS, protein–protein interactions (PPIs), and gene expression results. Network nodal weights were derived based on the p-value of each gene calculated through VEGAS2 (see genome-wide association analysis section). Edge weights (change of gene co-expression between death and alive) were obtained using the RNA-sequencing gene expression data. Genes within the top modules (0.1%) and also differentially methylated were selected (raw p < 0.05) and used for downstream pathway analysis using ConsensusPathDB^63^. Significant pathways were defined as q-value < 0.05.

#### Overlap gene approach

A loosened significance threshold (raw p < 0.05) was used to generate gene lists from each ‘omic’ including GWAS, RNA sequencing, and DNA methylation. Overlapping genes among these generated gene lists were used for downstream pathway analysis using ConsensusPathDB^63^. Significant pathways were defined as q-value < 0.05.

### Validation using longitudinal transcription analysis

Longitudinal transcription analysis of all genes within the specific pathway was performed to validate identified overlapping pathways through the *Network* and *Overlap* pathway approaches. The top significant pathways identified through the *network approach* were validated using the longitudinal transcription cohort (microarrays gene expression-derived data at baseline, Day 7, and Day 14). The longitudinal gene expression change over the 14 days was tested using the variance component score test^64^ using the R package *TcGSA*. This method is a linear mixed-effect model to account for repeat measurements from the same participants and a time variable is included. The pattern of gene expression changes in pathways differentially between ARDS non-survivors vs. survivors was tested with the null hypothesis that the patterns are the same between groups. Due to the small sample size (88 measurements), 1000 permutations were performed to calculate the p-value. The threshold for a significant difference in the patterns was set as an adjusted p-value < 0.05.

### Prediction model development

The transcription prediction model was developed using a logistic regression model with the genes identified (Table 2) through the *network approach* as biomarkers. We divided the baseline RNA microarray cohort into training and testing sets at a 7:3 ratio. The model was tested using the testing set and area under the receiver-operating characteristic curve (AUC). Each AUC with a 90% confidence interval was calculated. A good prediction model was defined as a model with AUC > 0.70 with a lower limit of confidence interval > 0.50.

For the protein prediction model, to have sufficient samples for the training, testing, and validation cohort, proteins were excluded if measured in less than 150 subjects. The remaining seven proteins including eNAMPT, IL-1β, IL-1R2, IL-8, Ang2, MIF, and S1PR3 were included as candidate biomarkers for the prediction model. We used a logistic regression model with the stepwise method. The chosen significance level for entry (SLE) and the chosen significance level for stay (SLS) was set at 0.25. We divided the randomly selected 190 subjects into training and testing sets at a 7:3 ratio respectively. The remaining 100 subjects were reserved as a validation cohort. We developed the models using baseline protein levels and Day 7 protein levels independently and then validated them. Each AUC with a 90% confidence interval was calculated. A good prediction model was defined as a model with AUC > 0.70 with a lower limit of confidence interval > 0.50. After the model development, we used the R package *cutpointr* to find the optimized threshold of the protein level to predict ARDS mortality.

## Supplementary Information

Supplementary Information.

## Data Availability

The datasets generated during and/or analyzed during the current study are available from the corresponding author on reasonable request and will also be deposited in the Gene Expression Omnibus (GEO176529).
