# Supplementary material for: Identification of early and intermediate biomarkers for ARDS mortality by multi-omic approaches
Source: Sci Rep. 2021 Sep 23;11:18874. doi: 10.1038/s41598-021-98053-1 (PMC8460799; doi:10.1038/s41598-021-98053-1)

**Supplement**

| **Table S1.** Top genes associated with ARDS mortality using gene-based analysis (raw P-value <0.01) | | | | |
| --- | --- | --- | --- | --- |
| Chromosome | Gene name | Start postion | Stop position | Pvalue |
| 2 | GPR75-ASB3 | 53847449 | 54137170 | 0.000625 |
| 2 | MIR3682 | 54026258 | 54126342 | 0.000658 |
| 2 | ASB3 | 53847116 | 54064146 | 0.000723 |
| 2 | ERLEC1 | 53964067 | 54095956 | 0.000845 |
| 17 | LLGL1 | 18078935 | 18198188 | 0.001004 |
| 1 | S100A10 | 1.52E+08 | 1.52E+08 | 0.001046 |
| 1 | S100A11 | 1.52E+08 | 1.52E+08 | 0.001077 |
| 1 | NBPF18P | 1.52E+08 | 1.52E+08 | 0.001223 |
| 2 | GPR75 | 54030049 | 54137170 | 0.00135 |
| 16 | TRAF7 | 2155798 | 2278130 | 0.001703 |
| 15 | NARG2 | 60661807 | 60821359 | 0.001714 |
| 17 | FLII | 18098130 | 18212230 | 0.001821 |
| 12 | C12orf79 | 92328751 | 92586447 | 0.001828 |
| 2 | PSME4 | 54041203 | 54247977 | 0.001948 |
| 2 | BOLA3 | 74312527 | 74425039 | 0.002812 |
| 12 | BTG1 | 92484053 | 92589673 | 0.003053 |
| 10 | PLAC9 | 81842257 | 81954784 | 0.003123 |
| 16 | SNORD60 | 2155023 | 2255106 | 0.003234 |
| 10 | ANXA11 | 81864879 | 82015433 | 0.003491 |
| 2 | CHAC2 | 53944928 | 54052287 | 0.003621 |
| 16 | RAB26 | 2148650 | 2254141 | 0.003767 |
| 9 | C9orf135-AS1 | 72384320 | 72485655 | 0.003825 |
| 2 | MIR3131 | 2.2E+08 | 2.2E+08 | 0.004068 |
| 5 | LYRM7 | 1.3E+08 | 1.31E+08 | 0.004093 |
| 3 | MIR922 | 1.97E+08 | 1.97E+08 | 0.004244 |
| 21 | KCNJ6 | 38946784 | 39338741 | 0.00455 |
| 5 | HINT1 | 1.3E+08 | 1.31E+08 | 0.004572 |
| 17 | MIEF2 | 18113847 | 18219095 | 0.004686 |
| 7 | TYW1 | 66411791 | 66754507 | 0.005016 |
| 1 | PPM1J | 1.13E+08 | 1.13E+08 | 0.005029 |
| 6 | MAP7 | 1.37E+08 | 1.37E+08 | 0.005057 |
| 17 | ALKBH5 | 18036866 | 18163267 | 0.005081 |
| 2 | IHH | 2.2E+08 | 2.2E+08 | 0.00513 |
| 2 | C2orf27B | 1.33E+08 | 1.33E+08 | 0.005559 |
| 1 | FAM19A3 | 1.13E+08 | 1.13E+08 | 0.005709 |
| 7 | EIF3B | 2344473 | 2470377 | 0.005735 |
| 2 | TET3 | 74163530 | 74385302 | 0.005887 |
| 9 | C9orf47 | 91555777 | 91661057 | 0.006286 |
| 7 | MIR4650-2_1 | 66529308 | 66629384 | 0.006361 |
| 4 | SMIM20 | 25865813 | 25981501 | 0.00639 |
| 9 | S1PR3 | 91556323 | 91670069 | 0.0065 |
| 7 | MIR4650-1_1 | 66529308 | 66629384 | 0.006523 |
| 12 | ZNF605 | 1.33E+08 | 1.34E+08 | 0.006664 |
| 8 | CHMP7 | 23051149 | 23169512 | 0.006683 |
| 16 | CASKIN1 | 2177183 | 2296465 | 0.006783 |
| 7 | MGC72080 | 97545907 | 97651638 | 0.006841 |
| 12 | LOC101928530 | 1.33E+08 | 1.34E+08 | 0.007239 |
| 1 | RBM15 | 1.11E+08 | 1.11E+08 | 0.007307 |
| 13 | FAM216B | 43305685 | 43415685 | 0.00743 |
| 7 | CHST12 | 2393194 | 2524216 | 0.007498 |
| 2 | MIR4429 | 11630730 | 11730803 | 0.007625 |
| 1 | LOC440600 | 1.11E+08 | 1.11E+08 | 0.007637 |
| 11 | MIR6124 | 12135229 | 12235314 | 0.007686 |
| 11 | PATE4 | 1.26E+08 | 1.26E+08 | 0.007745 |
| 7 | FAM180A | 1.35E+08 | 1.35E+08 | 0.007884 |
| 2 | RAPGEF4 | 1.74E+08 | 1.74E+08 | 0.008066 |
| 10 | FAM21C | 46172647 | 46338412 | 0.008125 |
| 17 | DLG4 | 7043209 | 7173369 | 0.008164 |
| 3 | CPB1 | 1.48E+08 | 1.49E+08 | 0.008237 |
| 11 | MRPL48 | 73448916 | 73625656 | 0.008402 |
| 9 | KIAA0020 | 2754154 | 2894130 | 0.008474 |
| 1 | RHOC | 1.13E+08 | 1.13E+08 | 0.008649 |
| 3 | CPA3 | 1.49E+08 | 1.49E+08 | 0.009073 |
| 8 | NECAB1 | 91753920 | 92021630 | 0.009369 |
| 14 | AJUBA_2 | 23397820 | 23501851 | 0.009518 |
| 7 | CHRM2 | 1.37E+08 | 1.37E+08 | 0.00965 |
| 12 | MIR6760 | 1.12E+08 | 1.12E+08 | 0.009708 |
| 19 | RAB8A | 16172489 | 16294445 | 0.009804 |
| 15 | LDHAL6B | 59449014 | 59550785 | 0.009914 |
| 11 | RTN4RL2 | 57178009 | 57295012 | 0.009958 |

| **Table S2.** Top genes associated with ARDS mortality using genome-wide RNA transcriptome analysis (raw P-value <0.001) | | |
| --- | --- | --- |
| Gene | Log fold change | P Value |
| PLK5 | 0.792298 | 2.29E-05 |
| AC092329.3 | -0.65541 | 7.11E-05 |
| ARHGEF33 | 1.369273 | 7.14E-05 |
| REG4 | -1.14709 | 0.000137 |
| TAS2R30 | -0.52547 | 0.000186 |
| ZNF521 | -0.89438 | 0.000215 |
| TRIM25 | 0.780646 | 0.000246 |
| BICDL2 | 2.039403 | 0.000267 |
| MTND3P12 | 1.041554 | 0.000293 |
| NLRP12 | 0.835591 | 0.000312 |
| MRVI1 | 1.533493 | 0.000331 |
| C5orf67 | 1.277534 | 0.000374 |
| NAP1L4P1 | 0.864659 | 0.000413 |
| NPIPB5 | -0.59034 | 0.000428 |
| LYVE1 | 1.934497 | 0.000452 |
| RPLP2 | -0.73638 | 0.000455 |
| NPIPB3 | -0.55185 | 0.000482 |
| RPL32 | -0.70125 | 0.000483 |
| AC099489.2 | 1.32969 | 0.000491 |
| PLXNC1 | 0.751985 | 0.00052 |
| CD55 | 0.736602 | 0.000521 |
| HIST2H2BF | 1.186101 | 0.000523 |
| AREL1 | 0.445417 | 0.000526 |
| IMPDH1P10 | 0.84989 | 0.000539 |
| CPD | 0.673786 | 0.000546 |
| AC021106.1 | 0.753467 | 0.000568 |
| TBC1D14 | 0.588535 | 0.00059 |
| P4HTM | -0.37545 | 0.000657 |
| SNAP23 | 0.314032 | 0.00066 |
| TMPRSS11B | 0.876314 | 0.000662 |
| H2AFB3 | 1.352623 | 0.000668 |
| TRBV13 | -1.03796 | 0.000694 |
| IL27 | 0.871029 | 0.000743 |
| MTCO3P23 | 1.081926 | 0.000766 |
| PRRT1 | -0.94017 | 0.000813 |
| HKR1 | -0.56329 | 0.000824 |
| TPM3P6 | -0.93648 | 0.000844 |
| CR1 | 1.032611 | 0.000857 |
| LILRA6 | 0.897976 | 0.000871 |
| SULT1B1 | 0.938582 | 0.000889 |
| ACSL4 | 0.589685 | 0.000952 |
| TLR8 | 0.698536 | 0.000968 |
| AC120114.5 | 0.838908 | 0.000968 |

| **Table S3.** Top methylation probe (CpG) associated with ARDS mortality (raw P-value <0.001) | | | | |
| --- | --- | --- | --- | --- |
| chr | Name | Gencode | logFC | P Value |
| chr1 | cg19741456 | FBXO6 | -0.36348 | 4.07E-06 |
| chr17 | cg25191743 | MAP3K14 | 0.303916 | 1.30E-05 |
| chr9 | cg14161705 |  | -0.25581 | 1.81E-05 |
| chr17 | cg05718652 | AC004448.5 | -0.13997 | 7.43E-05 |
| chr11 | cg26442852 |  | 0.25514 | 8.31E-05 |
| chr10 | cg24077454 | PDZD8 | 0.512831 | 8.37E-05 |
| chr12 | cg11302945 |  | -0.47227 | 8.65E-05 |
| chr7 | cg12973591 | TFPI2;AC002076.10;TFPI2;GNGT1 | -0.2463 | 0.000101 |
| chr2 | cg23333970 | STARD7;AC012307.3 | -0.377 | 0.000104 |
| chr20 | cg10003667 | GNAS | -0.1886 | 0.000119 |
| chr14 | cg05219996 | ZBTB25;ZBTB1 | -0.29554 | 0.000133 |
| chr8 | cg22879009 |  | 0.275106 | 0.000142 |
| chr3 | cg12727513 | SS18L2;SEC22C | 0.198421 | 0.000145 |
| chr11 | cg07044494 | FCHSD2 | 0.152385 | 0.000152 |
| chr16 | cg03194064 | DOK4 | -0.25134 | 0.000161 |
| chr20 | cg18438187 |  | 0.364214 | 0.000167 |
| chr19 | cg14052044 | C19orf21;C19orf21 | -0.32049 | 0.000177 |
| chr10 | cg14339664 | FAM13C | 0.191537 | 0.000187 |
| chr17 | cg21319411 | KRTAP4-1 | 0.240766 | 0.000192 |
| chr17 | cg25365746 | HIC1 | -0.23356 | 0.000193 |
| chr19 | cg11288278 | RYR1 | 0.266009 | 0.000198 |
| chr8 | cg27476810 | RB1CC1 | -0.21615 | 0.000202 |
| chr1 | cg17454086 | KIAA0907 | 0.317301 | 0.000213 |
| chr1 | cg07029998 | MTOR;MTOR | 0.172157 | 0.000217 |
| chr2 | cg23998726 | SNTG2 | 0.220972 | 0.000228 |
| chr17 | cg14210275 | TMEM107 | -0.27865 | 0.000233 |
| chr10 | cg08880064 |  | 0.153178 | 0.000235 |
| chr10 | cg21077330 | STK32C | 0.195783 | 0.000243 |
| chr4 | cg24987775 | FAM160A1 | 0.192262 | 0.000252 |
| chr14 | cg19715557 |  | 0.345358 | 0.000253 |
| chr2 | cg18481642 | STAT4 | 0.197439 | 0.000261 |
| chr7 | cg08164294 | HOXA3;HOXA-AS2 | 0.219219 | 0.000264 |
| chr3 | cg18729130 | SLC25A26;LRIG1 | 0.196612 | 0.000265 |
| chr11 | cg19364351 | CCKBR | -0.65995 | 0.000267 |
| chr17 | cg12259256 | TMEM101 | 0.155147 | 0.000282 |
| chr13 | cg05178518 | EXOSC8;ALG5 | -0.27577 | 0.000305 |
| chr4 | cg16781992 | KCNIP4 | 0.564027 | 0.000317 |
| chr11 | cg04467334 | CCKBR | -0.6337 | 0.000337 |
| chr1 | cg26483432 | HIST2H2AA3 | 0.23434 | 0.000341 |
| chr17 | cg12023999 | C17orf67 | 0.166267 | 0.000344 |
| chr16 | cg04556612 | DPEP3 | -0.19704 | 0.000358 |
| chr19 | cg00751958 | NLRP8 | -0.17861 | 0.00037 |
| chr2 | cg25654774 |  | 0.169898 | 0.000377 |
| chr20 | cg12933677 | VAPB | -0.30115 | 0.00038 |
| chr3 | cg13613891 | ZBTB47 | 0.273475 | 0.000384 |
| chr3 | cg13062627 |  | 0.213066 | 0.00039 |
| chr14 | cg27539233 |  | -0.40691 | 0.000395 |
| chr19 | cg26382679 |  | -0.44492 | 0.000398 |
| chr3 | cg17645664 | HTR3D | -0.13481 | 0.000412 |
| chr7 | cg26385074 |  | -0.38881 | 0.000421 |
| chr6 | cg01268704 |  | 0.401366 | 0.000428 |
| chr1 | cg23107691 | RBP7 | -0.36372 | 0.00043 |
| chr1 | cg16140432 | ZNF642 | -0.41369 | 0.000444 |
| chr1 | cg09135113 | TMEM48 | -0.25169 | 0.000448 |
| chr15 | cg15433343 | DENND4A;RAB11A | -0.26515 | 0.000451 |
| chr19 | cg24226087 | TYK2 | 0.162491 | 0.000457 |
| chr18 | cg21603500 | TRAPPC8 | -0.29465 | 0.000464 |
| chr22 | cg00704796 |  | -0.2447 | 0.000477 |
| chr18 | cg12240824 | ZNF271;ZSCAN30 | 0.12313 | 0.000493 |
| chr19 | cg04313968 | REXO1 | -0.14778 | 0.000499 |
| chr7 | cg16451475 |  | -0.50667 | 0.000503 |
| chr13 | cg01536987 | EPSTI1 | -0.40394 | 0.000506 |
| chr5 | cg06668065 | RP11-65F13.2 | 0.287176 | 0.00051 |
| chr6 | cg22483030 | HLA-DPB1 | -0.29126 | 0.000518 |
| chr19 | cg18084798 | RHPN2 | -0.20823 | 0.000525 |
| chr5 | cg19875969 | FAM172A | 0.403884 | 0.000528 |
| chr4 | cg05255728 | ZNF141 | 0.308766 | 0.000532 |
| chr7 | cg12660364 | ZMIZ2 | 0.370207 | 0.000532 |
| chr17 | cg17182270 |  | 0.157065 | 0.000544 |
| chr7 | cg12854762 | KIAA1549 | 0.337917 | 0.000552 |
| chr13 | cg14365785 | MTUS2-AS1;MTUS2 | 0.21285 | 0.000557 |
| chr6 | cg09989847 | NT5E | 0.268974 | 0.000559 |
| chr17 | cg02402274 | C17orf101 | 0.15086 | 0.000562 |
| chr6 | cg18644481 | EHMT2 | 0.256839 | 0.00057 |
| chr6 | cg21749424 |  | -0.63927 | 0.000622 |
| chr19 | cg15041373 |  | 0.235623 | 0.000632 |
| chr3 | cg03548224 | COL7A1 | 0.195337 | 0.000647 |
| chr1 | cg07176168 |  | -0.22593 | 0.000651 |
| chr1 | cg13562446 | RP11-296A18.3;RNF11 | -0.35012 | 0.000681 |
| chr6 | cg06178073 | ZNF391 | -0.32431 | 0.000684 |
| chr11 | cg26313599 | CCKBR | -0.64519 | 0.000685 |
| chr3 | cg03445587 | RP11-6F2.7;LEKR1 | -0.30513 | 0.000688 |
| chr2 | cg22745354 |  | -0.34215 | 0.000701 |
| chr4 | cg11552981 | KIAA1530 | 0.219761 | 0.000709 |
| chr7 | cg00696388 | TSPAN12 | -0.18629 | 0.000721 |
| chr7 | cg23756768 |  | -0.27232 | 0.000727 |
| chr16 | cg27453745 | FOXF1 | 0.263976 | 0.000731 |
| chr17 | cg03448527 | EIF4A3 | -0.18275 | 0.000732 |
| chr10 | cg09265586 | ADARB2-AS1 | 0.272344 | 0.000735 |
| chr1 | cg25306442 | ZSWIM5 | -0.21058 | 0.000761 |
| chr17 | cg16368008 | ASIC2 | -0.33018 | 0.000765 |
| chr11 | cg02335306 |  | 0.191098 | 0.000768 |
| chr1 | cg24467349 | GSTM5;RP4-735C1.4 | -1.05334 | 0.000768 |
| chr21 | cg01688776 | LINC00112;LINC00479;TMPRSS3 | -0.23751 | 0.000768 |
| chr16 | cg16720944 |  | -0.17448 | 0.000785 |
| chr20 | cg25840824 | PMEPA1 | 0.159938 | 0.000788 |
| chr5 | cg14725858 | NADKD1 | -0.16908 | 0.000792 |
| chr16 | cg01300778 | C16orf72 | 0.333483 | 0.000798 |
| chr5 | cg15925343 |  | 0.203036 | 0.000802 |
| chr15 | cg13617889 |  | -0.1819 | 0.000804 |
| chr2 | cg06024411 | FBLN7 | -0.39997 | 0.000814 |
| chr3 | cg25929976 | PPARG | -0.37768 | 0.000816 |
| chr15 | cg11520554 | ST20;C15orf37;AC015871.2;RP11-38G5.1 | -0.26834 | 0.000818 |
| chr16 | cg08390099 | RMI2 | 0.235436 | 0.000828 |
| chr10 | cg15988457 |  | 0.191647 | 0.000842 |
| chr7 | cg14990857 | TRIM74;STAG3L3 | -0.23471 | 0.000843 |
| chr14 | cg16097124 | TMED10 | 0.320821 | 0.000845 |
| chr21 | cg11705651 | UBE2G2;SUMO3 | 0.269963 | 0.000848 |
| chr19 | cg10769313 |  | -0.27151 | 0.00085 |
| chr2 | cg15016481 | LRRFIP1 | 0.165865 | 0.000866 |
| chr1 | cg08130307 |  | 0.156791 | 0.00087 |
| chr1 | cg04097724 |  | -0.19625 | 0.000873 |
| chr8 | cg17779658 | AC145110.1;C8orf75 | -0.37551 | 0.000876 |
| chr11 | cg27094173 | PLEKHB1 | -0.20753 | 0.00088 |
| chr13 | cg00012692 | GJB6 | -0.17906 | 0.000892 |
| chr2 | cg17836141 |  | -0.4425 | 0.000896 |
| chr6 | cg23500637 |  | 0.323932 | 0.000899 |
| chr9 | cg13763482 | PPAPDC2;SPATA6L | 0.4023 | 0.000899 |
| chr2 | cg09581060 |  | -0.32781 | 0.000911 |
| chr11 | cg24197212 | RPS6KB2 | -0.28717 | 0.00092 |
| chr8 | cg14737877 | ERICH1 | -0.25402 | 0.000934 |
| chr11 | cg04647918 |  | -0.45859 | 0.00094 |
| chr10 | cg17422176 | C10orf105;CDH23 | -0.35952 | 0.000942 |
| chr11 | cg04906985 | MRPL48 | 0.190762 | 0.000945 |
| chr17 | cg16011583 | C17orf82 | 0.279668 | 0.000949 |
| chr10 | cg12758503 | ZWINT | 0.162605 | 0.00096 |
| chr10 | cg13637321 | MXI1 | -0.37906 | 0.000962 |

| **Table S4.** Associations between the protein level and ARDS mortality | | | |
| --- | --- | --- | --- |
|  | Estimate | SE | p-value |
| **Baseline protein** |  |  |  |
| eNAMPT | 4.09E-04 | 1.53E-03 | 0.788587 |
| IL1B | 0.002209 | 0.002218 | 3.19E-01 |
| IL1R2 | -9.33E-07 | 4.70E-06 | 8.43E-01 |
| IL6 | -4.51E-05 | 9.80E-05 | 6.46E-01 |
| IL8 | -0.00021 | 0.000225 | 3.50E-01 |
| VEGF | -0.00015 | 0.001662 | 0.927706 |
| Ang2 | -0.00619 | 0.006237 | 3.21E-01 |
| MIF | -6.12E-05 | 0.000578 | 9.16E-01 |
| S1PR3 | 0.00017 | 0.000127 | 1.81E-01 |
| RAGE | -3.46E-06 | 9.88E-05 | 0.97204 |
| HMGB1 | -0.00071 | 0.000594 | 0.230918 |
| **Day 7 protein** |  |  |  |
| eNAMPT | -0.00031 | 0.001288 | 8.12E-01 |
| IL1B | -0.00254 | 0.003631 | 4.84E-01 |
| IL1R2 | 1.62E-05 | 1.15E-05 | 1.60E-01 |
| IL6 | 0.001089 | 0.001161 | 3.48E-01 |
| IL8 | 0.000802 | 0.000421 | 5.66E-02 |
| VEGF | 0.001697 | 0.002694 | 0.528922 |
| Ang2 | 0.076645 | 0.016738 | 4.67E-06 |
| MIF | 0.002302 | 0.001695 | 1.74E-01 |
| S1PR3 | 4.7E-05 | 3.66E-05 | 2.00E-01 |
| RAGE | -9.46E-05 | 0.000424 | 0.823329 |
| HMGB1 | -5.11E-05 | 0.000199 | 0.797702 |

| **Table S5:** Summary of the potential biomarkers identified using a multi-omics approach.^‡^ | | | | | | | |
| --- | --- | --- | --- | --- | --- | --- | --- |
| Omics Data | | GWAS data | RNA sequencing | | Differential Methylation | | |
| Analysis | | Gene-based analysis | Differential expression analysis | |  | | |
| Gene name*^†^* | Chr | p-value | LogFC | p-value | Probe | LogFC | p-value* |
| TNPO1 | 5 | 0.45 | -0.01 | 0.90 | cg12026956 | -0.10 | 0.04 |
| NUP214 | 9 | 0.21 | 0.03 | 0.83 | cg09553448  cg21213111 | 0.33  0.19 | 0.01  0.04 |
| HDAC1 | 1 | 0.17 | -0.15 | 0.11 | cg23698287  cg18857467 | 0.11  0.25 | 0.02  0.04 |
| HNRNPA1 | 12 | 0.09 | -0.56 | 0.12 | cg09158487  cg24505978 | -0.34  0.09 | 0.01  0.03 |
| GATAD2A | 19 | 0.11 | 0.07 | 0.41 | cg19057459  cg18520411 | 0.14  0.11 | 0.01  0.04 |
| FOSB | 19 | 0.02 | 0.29 | 0.56 | cg06941879 | 0.09 | 0.04 |
| DDX17 | 22 | 0.14 | -0.26 | 0.10 | cg08522428 | -0.24 | 0.004 |
| PHF20 | 20 | 0.90 | 0.10 | 0.28 | cg12579300 | -0.11 | 0.03 |
| CREBBP | 16 | 0.35 | 0.08 | 0.44 | cg06614534  cg03039399 | -0.15  -0.28 | 0.03  0.01 |
| Abbreviations: GWAS: genome-wide association study; Chr: chromosome; LogFC: log of the fold change  *Only reported the methylation results with raw p-values <0.05  ^†^Novel genes were genes identified through a *network approach* (Genes in the top 0.1% modules among the 5,854 modules and also overlap with differential methylation analysis)  ^‡^No protein level was measured on the novel genes identified through a *network approach* | | | | | | | |

| **Table S6.** Significant pathways associated with ARDS mortality using the *Network approach*. | | |
| --- | --- | --- |
| pathway | source | q-value |
| Signaling events mediated by HDAC Class I | PID | 3.45E-06 |
| TGF-beta Signaling Pathway | Wikipathways | 5.55E-05 |
| TGF_beta_Receptor | NetPath | 0.000108936 |
| IL6 | NetPath | 0.000326876 |
| Chromatin modifying enzymes | Reactome | 0.000326876 |
| Chromatin organization | Reactome | 0.000326876 |
| sumoylation by ranbp2 regulates transcriptional repression | BioCarta | 0.00049689 |
| Sumoylation by RanBP2 regulates transcriptional repression | PID | 0.000501488 |
| SUMO E3 ligases SUMOylate target proteins | Reactome | 0.000601835 |
| SUMOylation | Reactome | 0.000601835 |
| Transcriptional Regulation by TP53 | Reactome | 0.000601835 |
| Estrogen-dependent gene expression | Reactome | 0.00073564 |
| ESR-mediated signaling | Reactome | 0.000800946 |
| Regulation of TP53 Activity | Reactome | 0.000958368 |
| multi-step regulation of transcription by pitx2 | BioCarta | 0.000958368 |
| Retinoic acid receptors-mediated signaling | PID | 0.000972434 |
| Regulation of TP53 Activity through Acetylation | Reactome | 0.000972434 |
| wnt signaling pathway | BioCarta | 0.000981396 |
| Initiation of transcription and translation elongation at the HIV-1 LTR | Wikipathways | 0.00099137 |
| Signaling by Nuclear Receptors | Reactome | 0.001141988 |
| Regulation of mRNA stability by proteins that bind AU-rich elements | Reactome | 0.001202215 |
| IL-6 signaling pathway | Wikipathways | 0.001468659 |
| Notch Signaling | Wikipathways | 0.001468659 |
| Presenilin action in Notch and Wnt signaling | PID | 0.001468659 |
| NOTCH1 Intracellular Domain Regulates Transcription | Reactome | 0.001468659 |
| Notch-mediated HES/HEY network | PID | 0.001468659 |
| Hedgehog signaling events mediated by Gli proteins | PID | 0.001468659 |
| Notch signaling pathway - Homo sapiens (human) | KEGG | 0.001468659 |
| RNA Polymerase I Transcription Initiation | Reactome | 0.001468659 |
| Regulation of Androgen receptor activity | PID | 0.001540439 |
| Constitutive Signaling by NOTCH1 PEST Domain Mutants | Reactome | 0.001540439 |
| Signaling by NOTCH1 PEST Domain Mutants in Cancer | Reactome | 0.001540439 |
| Constitutive Signaling by NOTCH1 HD+PEST Domain Mutants | Reactome | 0.001540439 |
| Signaling by NOTCH1 HD+PEST Domain Mutants in Cancer | Reactome | 0.001540439 |
| Signaling by NOTCH1 in Cancer | Reactome | 0.001540439 |
| Role of Calcineurin-dependent NFAT signaling in lymphocytes | PID | 0.001610568 |
| Regulation of PTEN gene transcription | Reactome | 0.001920055 |
| Regulation of retinoblastoma protein | PID | 0.001940606 |
| Validated nuclear estrogen receptor alpha network | PID | 0.001940606 |
| Amphetamine addiction - Homo sapiens (human) | KEGG | 0.002134915 |
| SUMOylation of chromatin organization proteins | Reactome | 0.00220652 |
| Signaling by NOTCH1 | Reactome | 0.002322727 |
| Gene expression (Transcription) | Reactome | 0.002322727 |
| E2F transcription factor network | PID | 0.002358327 |
| Regulation of nuclear SMAD2/3 signaling | PID | 0.002429647 |
| ERCC6 (CSB) and EHMT2 (G9a) positively regulate rRNA expression | Reactome | 0.0024385 |
| Glucocorticoid receptor regulatory network | PID | 0.002509586 |
| Androgen receptor signaling pathway | Wikipathways | 0.003035348 |
| Formation of the beta-catenin:TCF transactivating complex | Reactome | 0.003243597 |
| HDACs deacetylate histones | Reactome | 0.00324666 |
| PTEN Regulation | Reactome | 0.003318243 |
| Cellular response to heat stress | Reactome | 0.003458381 |
| Positive epigenetic regulation of rRNA expression | Reactome | 0.004028473 |
| RNA Polymerase I Promoter Clearance | Reactome | 0.004173131 |
| RNA Polymerase I Transcription | Reactome | 0.004243863 |
| TGF-beta super family signaling pathway canonical | INOH | 0.004311613 |
| Thyroid hormone signaling pathway - Homo sapiens (human) | KEGG | 0.004311613 |
| Signaling by NOTCH | Reactome | 0.004455445 |
| Cell cycle - Homo sapiens (human) | KEGG | 0.004748796 |
| HATs acetylate histones | Reactome | 0.006090484 |
| Disease | Reactome | 0.006172532 |
| Epigenetic regulation of gene expression | Reactome | 0.006479977 |
| Generic Transcription Pathway | Reactome | 0.006479977 |
| Sudden Infant Death Syndrome (SIDS) Susceptibility Pathways | Wikipathways | 0.007120532 |
| Cell Cycle | Reactome | 0.007737866 |
| Metabolism of RNA | Reactome | 0.008444206 |
| Alcoholism - Homo sapiens (human) | KEGG | 0.00865776 |
| RNA Polymerase II Transcription | Reactome | 0.008977135 |
| TCF dependent signaling in response to WNT | Reactome | 0.009146484 |
| Huntington disease - Homo sapiens (human) | KEGG | 0.009485983 |
| Transcriptional regulation by RUNX1 | Reactome | 0.009635738 |
| Viral carcinogenesis - Homo sapiens (human) | KEGG | 0.009976179 |
| Ciliary landscape | Wikipathways | 0.010954659 |
| PIP3 activates AKT signaling | Reactome | 0.010954659 |
| TNFalpha | NetPath | 0.012835678 |
| Processing of Capped Intron-Containing Pre-mRNA | Reactome | 0.013297349 |
| Intracellular signaling by second messengers | Reactome | 0.013653761 |
| Diseases of signal transduction | Reactome | 0.013796588 |
| Signaling by WNT | Reactome | 0.016708986 |
| MicroRNAs in cancer - Homo sapiens (human) | KEGG | 0.019210215 |
| Signal Transduction | Reactome | 0.022952346 |
| Human papillomavirus infection - Homo sapiens (human) | KEGG | 0.023756 |
| Cellular responses to stress | Reactome | 0.024124708 |
| Cellular responses to external stimuli | Reactome | 0.033530808 |
| EGFR1 | NetPath | 0.039622337 |
| Cell Cycle, Mitotic | Reactome | 0.043196616 |
| Pathways in cancer - Homo sapiens (human) | KEGG | 0.050440767 |

**Figure S1.** Venn diagram for sample size and omics overlaps. Each color represented each ‘omic’ type. Genetic represented subjects with GWAS data; Transcriptions represented subjects with gene expression data (either RNA-sequencing or micro-array); Methylations represented subjects with methylation data (either 450K or EPIC 850K array); Proteins represented subjects with protein measurement (any protein measurement).


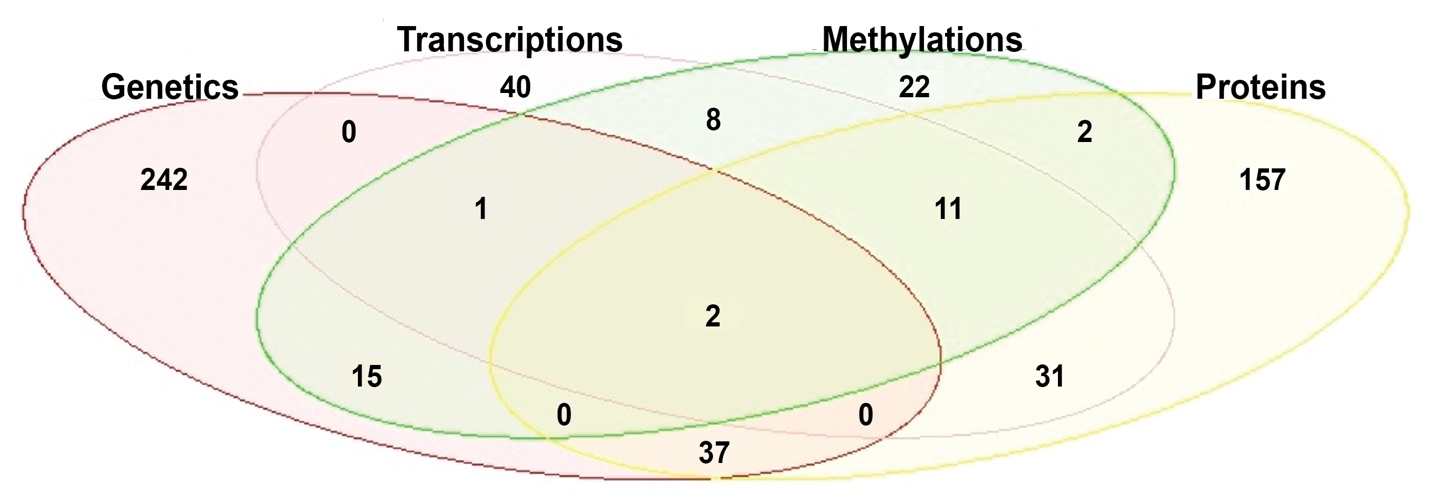


**Figure S2.** Bioinformatic analysis flow chart. Different color represents the different ‘omic’ types. Arrows mean the direction of the analysis flow. Text (not bold) represents the statistical tests. ED= European Descent, AD= African Descent


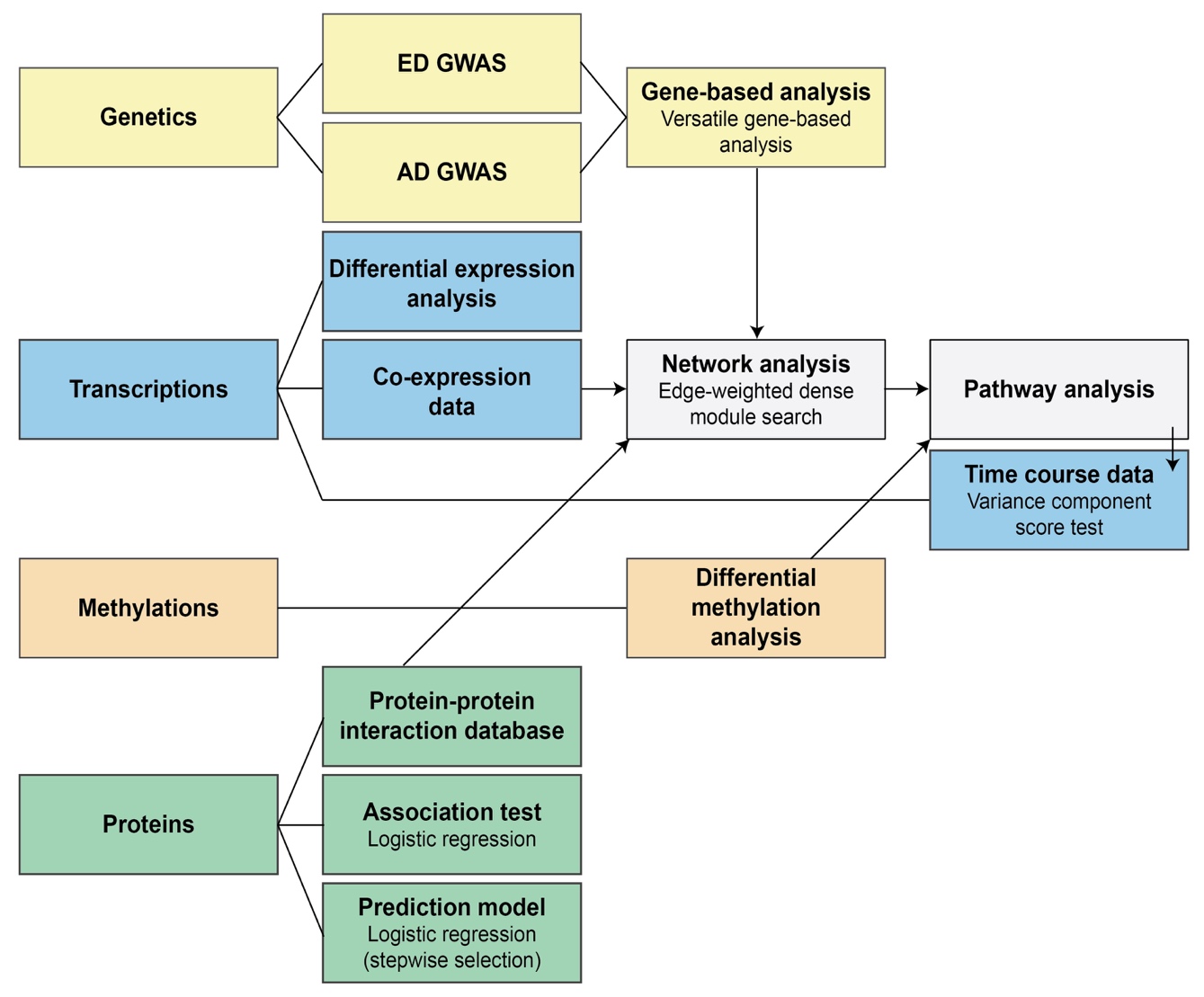

Supplement: Supplementary file 1 — Supplementary Information. [file 41598_2021_98053_MOESM1_ESM.docx]
